# Supplementary material for: A mutational hotspot that determines highly repeatable evolution can be built and broken by silent genetic changes
Source: Nat Commun. 2021 Oct 19;12:6092. doi: 10.1038/s41467-021-26286-9 (PMC8526746; doi:10.1038/s41467-021-26286-9)
Supplement: Supplementary file 1 — Supplementary Information [file 41467_2021_26286_MOESM1_ESM.pdf]

Supplementary Information

Methods:

Assessing Pleiotropy via Growth Rate

Cryopreserved samples of AR2 and derived *ntrB* mutants were streaked and grown for 48 h on LB agar (1.5%). Three colonies were then picked, inoculated in LB broth and grown overnight at an agitation of 180 rpm to create biological triplicates for each sample. This process was repeated with an independent batch of biological triplicates on a separate day to produce a total of 6 biological replicates for each sample. Overnight cultures were pelleted via centrifugation, their supernatant withdrawn and the cell pellets re-suspended in phosphate buffer saline (PBS) to a final concentration of OD1 cells/ml. The resuspension was subsequently diluted 100-fold into a 96-well plate (Costar®) containing nutrient broth. The plates were analysed in a Multiskan™ FC Microplate Photometer (Thermo Fisher Scientific) for 24h, with autonomous OD readings every 10 min without agitation. Growth values were determined by calculating area under the curve using the trapezoidal rule (approach outlined in[1]). This allowed us to incorporate elements of the pleiotropic consequences to metabolism as well as the benefits of the motile swimming phenotype, including prolonged lag phases, steeper exponential phases and differing eventual yields achieved by mutant populations relative to the ancestral strain (growth curves not shown).

ntrB loci analysis

Theoretical hairpin stem-loop structures were generated using the *mfg* tool and methodology developed by Wright *et al.*,[2]. The *mfg* tool is used in conjunction with the Quikfold tool on the DINAMelt Web Server[3]. Default parameters were used for Quikfold with the exception of temperature, which was amended to 27^{0}C. The first 400 nucleotides of the open reading frames of *P. fluorescens* SBW25 *ntrB* and Pf0-1 *ntrB* were used as input sequences, and AR2-sm's and Pf0-2x-sm's input sequences were created by manually editing SBW25's and Pf0-1's *ntrB* sequence. The *mfg* application generates the most stable stem-loop structure for each base in which the selected base remains unpaired and so is at a higher likelihood of mutation. The window size of neighbouring nucleotides that are used to form the stem-loop structure can be adjusted, and a window length of 40 nucleotides was used for the analysis in this study.

Figures and figure legends:

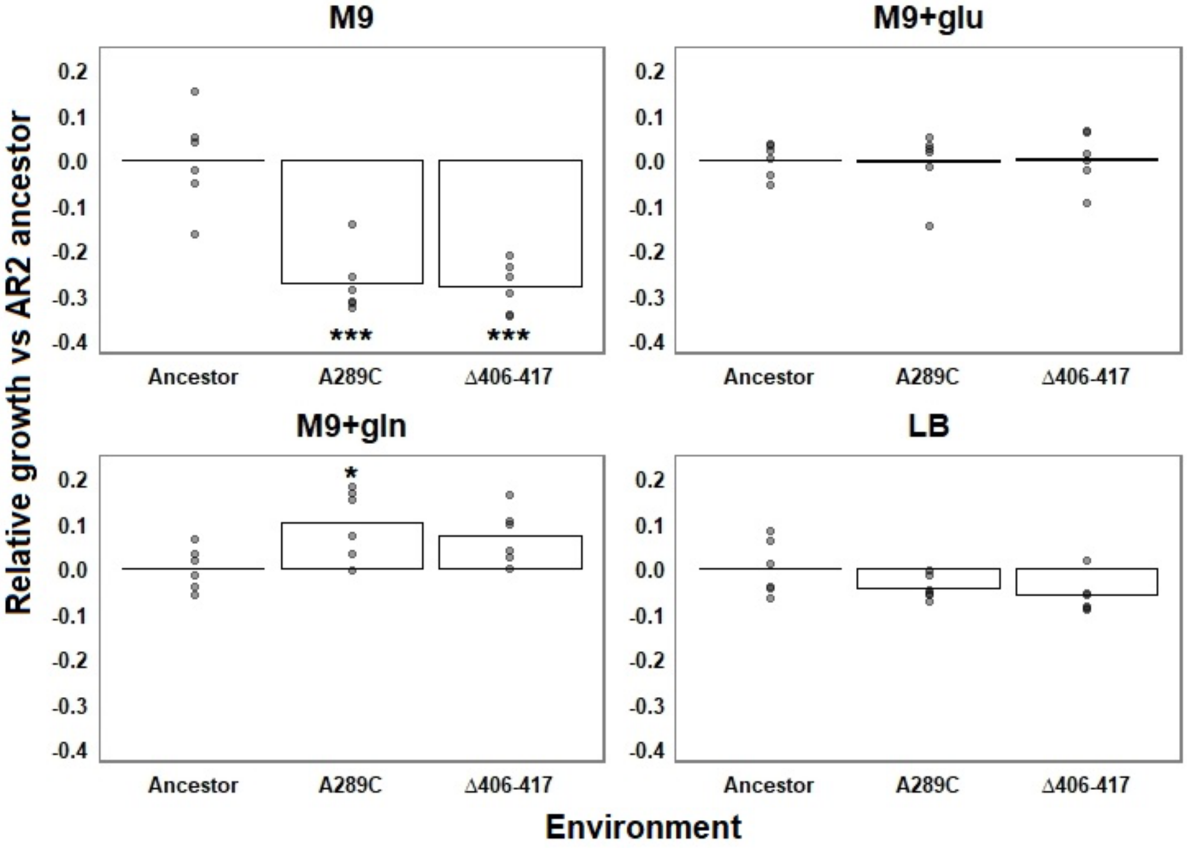

Supplementary Fig. 1. Growth kinetics of mutant AR2 lines in static liquid culture over 24h reveals environment-dependent pleiotropy. Nutrient environments: M9 = M9 minimal media supplemented with NH_4 at 7.5 mM. M9+glu = additional glutamate added at 8 mM. M9+gln = additional glutamine added at 8 mM. LB = lysogeny broth (*N* = 6 independent biological replicates per strain for all conditions). Growth yield was determined using area under the curve, and each yield has been standardised against the yield of the AR2 ancestral strain grown in the same environment (AR2 ancestor growth mean = 0). Individual data points from biological replicates are plotted, and ranges around the mean growth of the ancestral strain are shown in column one of each frame. Plots are the means of six biological replicates. Significance values for M9: A289C *P* = 0.000079, Δ406-417 *P* = 0.000056, for M9+gln: A289C *P* = 0.036 (one-way ANOVA post-hoc Tukey HSD test). Source data are provided as a Source Data file.

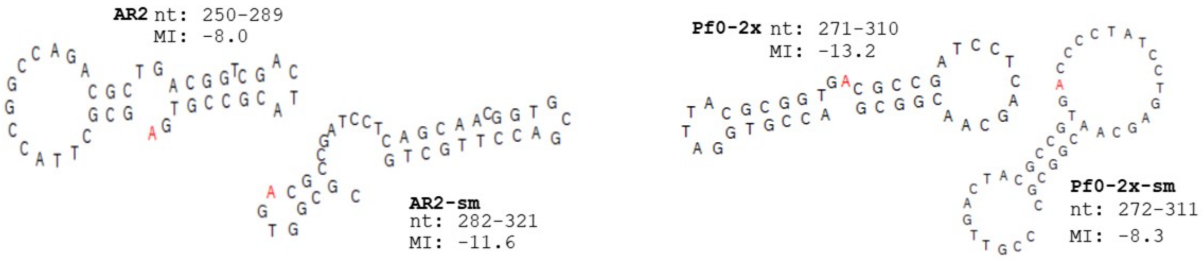

Supplementary Fig. 2. Quasi-palindromic sequences flank *ntrB* site 289 in both *P. fluorescens* SBW25 and Pf0-1 derived strains. Theoretical hairpin formations were generated using the *mfg* program[2]. This software calculates the most stable hairpin formed between neighbouring tracts (± 40 nucleotides from site 289) in which the site of interest (in this case site 289, highlighted in red) remains unpaired. In these examples the nucleotides are forced into stem-loop structures that have been documented to comprise hairpins[4]. The stability, structure and included nucleotide tracts of the predicted hairpins differ between strains and determine the mutated nucleotide site's Mutational Index (MI), which is a multiplication of the secondary structure's maximum energy (ΔG) and the percentage of alternative DNA folds in which the base of interest is unpaired: AR2 = -8.0. AR2-sm = -11.6, Pf0-2x = -13.2, Pf0-2x-sm(6) = -8.3.These differences are partially owed to synonymous sequence variation as highlighted by the altered hairpin formation exhibited by AR2-sm and Pf0-2x-sm, which differ from their ancestors by 6 synonymous substitutions. AR2 and Pf0-2x-sm, the two strains that evolve in a highly parallel manner, share similar features that are absent in the other two strains. Namely their MI's are similar (-8.0 and -8.3) and the frequently mutated 'A' is located two nucleotides away from the base of a singular long, stable stem. As the *mfg* program only calls the most stable hairpin configuration it may miss alternative structures that temporarily form and introduce mutation bias, however the tool exemplifies the power of synonymous variance in altering hairpin stability.

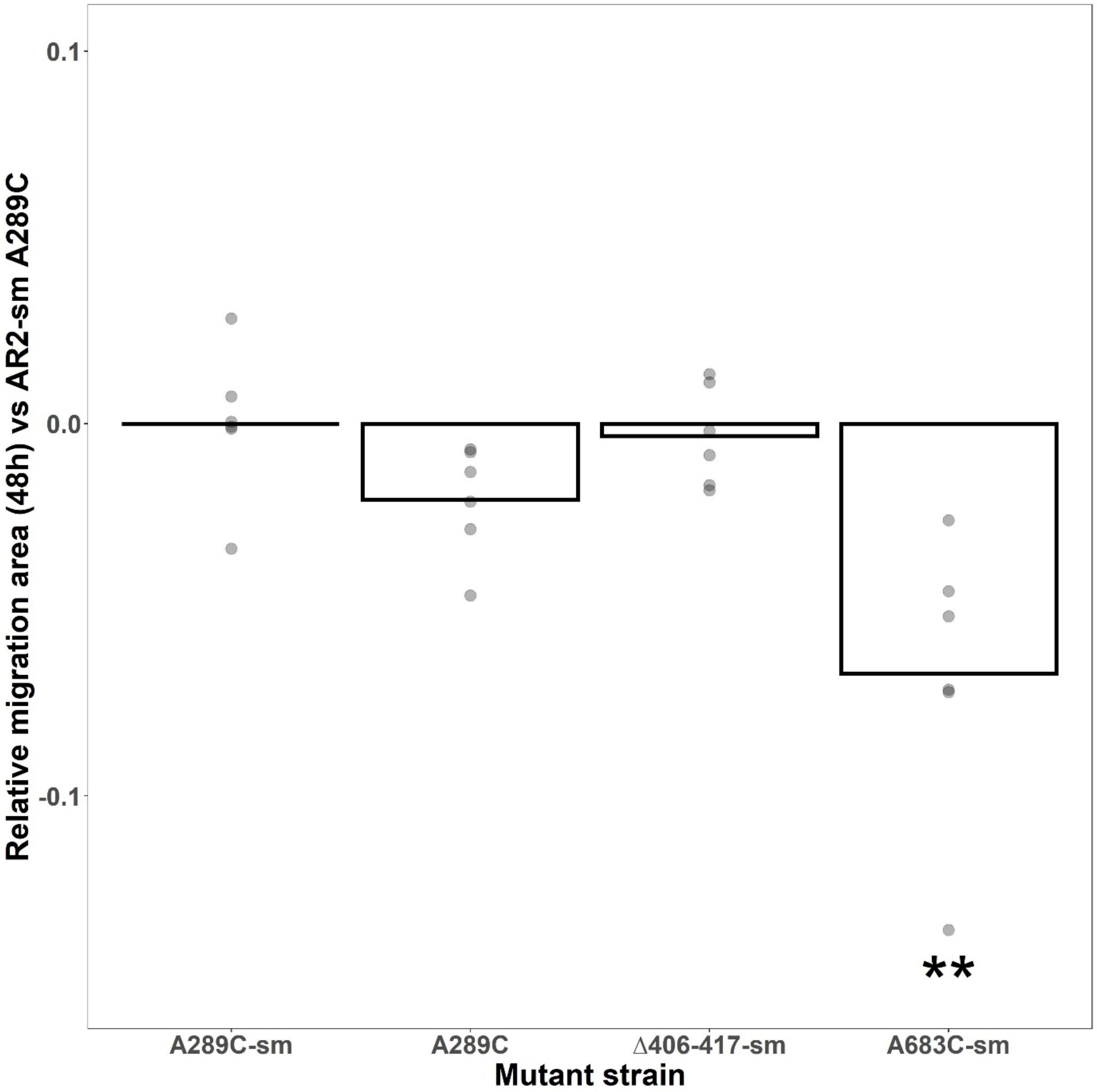

Supplementary Fig. 3. *ntrB* A289C in AR2-sm retains comparative fitness to its ancestral counterpart. The motility phenotype of AR2 *ntrB* A289C ($N = 5$ independent biological replicates) and alternative AR2-sm *ntrB* mutants (Δ406-417-sm ($N = 6$) and A683-sm ($N = 6$)) were measured against an engineered AR2-sm *ntrB* A289C mutant (A289C-sm ($N = 6$)) in minimal medium. A289C-sm was not significantly outperformed by any strain, instead showing a significantly superior motility phenotype to A683-sm in M9. Although the two motile lines displayed comparable motility in an AR2 background (Fig. 3), the inferior phenotype observed here may be owed to an uncharacterised secondary mutation. Individual data points from biological replicates are plotted and each migration area has been standardised against the surface area of a *ntrB* A289C-sm mutant grown in the same environment (*ntrB* A289C-sm growth mean = 0). Significance values for A683-sm: $P = 0.0036$, Kruskal-Wallis post-hoc Dunn test, one-sided). Source data are provided as a Source Data file.

Supplementary Table 1. List of primers used throughout the study.

For use in:	Primer description:	Sequence:
Sanger sequencing of ntr pathway / Invasion assay	SBW25 *ntrB* locus (forward)	5'- GAGGTCCCAATGACCATCAG -3'
	SBW25 *ntrB* locus (reverse)	5'- GACGATCCAGACGGTTTCAC -3'
	SBW25 *glnK* locus (forward)	5'-GTGGGCAAAGGACTGATTTC-3'
	SBW25 *glnK* locus (reverse)	5'-GATGATGGCGAAGGTCATCT-3'
	SBW25 *glnA* locus (forward)	5'-CGGAAATCGCTCAAGGTTTA-3'
	SBW25 *glnA* locus (reverse)	5'-CTGATAATCCCCAGGCAAAA-3'
AR2 *ntrB* A683C integration into pTS1 backbone (allelic exchange)	Upstream fragment (forward)	5'- GAAATTAATAGGTTGTATTGATGTTGATGACCATCAGCGATGCACTG -3'
	Upstream fragment (reverse)	5'- GAATGCTCGGGGCGTAGTCGC -3'
	Downstream fragment (forward)	5'- GCGACTACGCCCCGAGCATTC -3'
	Downstream fragment (reverse)	5'- GCCGTTTCTGTAATGAAGGAGAAAACTCATGTCGATGGGGCTCCTTG -3'
AR2 *ntrB* synonymous substitution sequence integration into pTS1 backbone (allelic exchange)	Upstream fragment (forward)	5'- GAAATTAATAGGTTGTATTGATGTTGTGCCAAATGCCGCCTACATC -3'
	Upstream fragment (reverse)	5'- CGTTGCTGAGGATCGGCGTCACCGCGTAATCCACCGTCAG -3'
	Downstream fragment (forward)	5'- CTGACGGTGGATTACGCGGTGACGCCGATCCTCAGCAACG -3'
	Downstream fragment (reverse)	5'- GCCGTTTCTGTAATGAAGGAGAAAACGTTGATCAGCACGGTGATGT -3'
	SBW25 *ntrB* nested primer (forward)	5'- AATTTGGATCCATGACCATCAGCGATGCACTG -3'
	SBW25 *ntrB* nested primer (reverse)	5'- AATTTAAGCTTGATCCAGACGGTTTCACTACG -3'
AR2 *ntrB* synonymous substitution sequence with A289C	Upstream fragment (reverse)	5'- CGTTGCTGAGGATCGGCGGCACCGCGTAATCCACCGTCAG -3'
	Downstream fragment (forward)	5'- CTGACGGTGGATTACGCGGTGCCGCCGATCCTCAGCAACG -3'
Pf0-2x *ntrB* synonymous substitution sequence integration into pTS1 backbone (allelic exchange)	Upstream fragment (forward)	5'-TATCGCCTGCTGCTGGATGG-3'
	Upstream fragment (reverse)	5'- CGTTGCTCAGGATAGGGGTCACGGCGTAGTCGACGGTCAG -3'
	Downstream fragment (forward)	5'- CTGACCGTCGACTACGCCGTGACCCCTATCCTGAGCAACG -3'
	Downstream fragment (reverse)	5'-TCCACACGGTTTCACTACGG-3'
	Pf0-1 *ntrB* nested primer (forward)	5'-AATTTGGATCCAGCGTCAGGTCAAACCGTGT-3'
	Pf0-1 *ntrB* nested primer (reverse)	5'-AATTTAAGCTTTGGTGCTGGCTGATGATGTT-3'
Screening engineered lines for counter-selection escape	*sacB* check (Forward)	5'-TCAATCATACCGAGAGCGCC-3'
	sacB check (Reverse)	5'-TGTCGCAAACTATCACGGCT-3'

References:

1. Huang, S. & Pang, L. Comparing statistical methods for quantifying drug sensitivity based on in vitro dose-response assays. *Assay Drug Dev. Technol.* **10**, 88–96 (2012).

2. Wright, B. E., Reschke, D. K., Schmidt, K. H., Reimers, J. M. & Knight, W. Predicting mutation frequencies in stem-loop structures of derepressed genes: Implications for evolution. *Mol. Microbiol.* **48**, 429–441 (2003).

3. Markham, N. R. & Zuker, M. DINAMelt web server for nucleic acid melting prediction. *Nucleic Acids Res.* **33**, 577–581 (2005).

4. Ripley, L. S. Model for the participation of quasi-palindromic DNA sequences in frameshift mutation. *Proc. Natl. Acad. Sci. U. S. A.* **79**, 4128–4132 (1982).
