# Supplementary material for: A mutational hotspot that determines highly repeatable evolution can be built and broken by silent genetic changes
Source: Nat Commun. 2021 Oct 19;12:6092. doi: 10.1038/s41467-021-26286-9 (PMC8526746; doi:10.1038/s41467-021-26286-9)
Supplement: Supplementary file 3 — Reporting Summary [file 41467_2021_26286_MOESM3_ESM.pdf]

nature research

Corresponding author(s): James S. Horton and Tiffany B. Taylor

Last updated by author(s): Aug 3, 2021

Reporting Summary

Nature Research wishes to improve the reproducibility of the work that we publish. This form provides structure for consistency and transparency in reporting. For further information on Nature Research policies, see our Editorial Policies and the Editorial Policy Checklist.

Statistics

For all statistical analyses, confirm that the following items are present in the figure legend, table legend, main text, or Methods section.

n/a	Confirmed	
☐	☒	The exact sample size (*n*) for each experimental group/condition, given as a discrete number and unit of measurement
☐	☒	A statement on whether measurements were taken from distinct samples or whether the same sample was measured repeatedly
☐	☒	The statistical test(s) used AND whether they are one- or two-sided<br>*Only common tests should be described solely by name; describe more complex techniques in the Methods section.*
☐	☒	A description of all covariates tested
☐	☒	A description of any assumptions or corrections, such as tests of normality and adjustment for multiple comparisons
☐	☒	A full description of the statistical parameters including central tendency (e.g. means) or other basic estimates (e.g. regression coefficient) AND variation (e.g. standard deviation) or associated estimates of uncertainty (e.g. confidence intervals)
☒	☐	For null hypothesis testing, the test statistic (e.g. *F*, *t*, *r*) with confidence intervals, effect sizes, degrees of freedom and *P* value noted<br>*Give P values as exact values whenever suitable.*
☒	☐	For Bayesian analysis, information on the choice of priors and Markov chain Monte Carlo settings
☒	☐	For hierarchical and complex designs, identification of the appropriate level for tests and full reporting of outcomes
☒	☐	Estimates of effect sizes (e.g. Cohen's *d*, Pearson's *r*), indicating how they were calculated

Our web collection on statistics for biologists contains articles on many of the points above.

Software and code

Policy information about availability of computer code

Data collection	Single nucleotide polymorphisms (SNPs) and small insertions/deletions (indels) were called genome-wide using Snippy version 4.3.8 (Seemann, T. Snippy: fast bacterial variant calling from NGS reads. (2015)). SNPs and indels were called from Sanger sequencing data using nucleotide BLAST (Altschul et al., Basic local alignment search tool. (1990). J. Mol. Biol. 215:403-410). We determined the proportion of synonymous vs non-synonymous nucleotide changes in the ntrB locus between two strains of P. fluorescens using custom code and BLAST. The custom code is available with a readme instruction file on GitHub (https://github.com/J-S-Horton/Syn-sequence-parallel-evolution).
Data analysis	Theoretical stem-loop structures were generated using the mfg tool version 1.2, written and reported by Wright et al., 2003 (Wright BE, Reschke DK, Schmidt KH, Reimers JM, Knight W. 2003. Predicting mutation frequencies in stem-loop structures of derepressed genes: Implications for evolution. Mol. Microbiol. 48:429–441) using ntrB sequences downloaded from the Pseudomonas Genome Database (https://www.pseudomonas.com/). All statistical tests and figures were produced in R version 3.6.0 (R Core Team. 2014. R: A language and environment for statistical computing. R Found. Stat. Comput. Vienna, Austria. Available from: http://www.r-project.org/). All tests were completed using functions in base-R aside from the Dunn test, which was performed using the FSA package (Ogle DH, Wheeler P, Dinno A. 2020. FSA: Fisheries Stock Analysis. Available from: https://github.com/droglenc/FSA). Figures were created using the ggplot package (Wickham H. 2016. ggplot2: Elegant Graphics for Data Analysis. Available from: https://ggplot2.tidyverse.org).

For manuscripts utilizing custom algorithms or software that are central to the research but not yet described in published literature, software must be made available to editors and reviewers. We strongly encourage code deposition in a community repository (e.g. GitHub). See the Nature Research guidelines for submitting code & software for further information.

Data

Policy information about availability of data

All manuscripts must include a data availability statement. This statement should provide the following information, where applicable:

- Accession codes, unique identifiers, or web links for publicly available datasets
- A list of figures that have associated raw data
- A description of any restrictions on data availability

The data used in this study are available in the Open Science Framework database, accessible via the following link: https://osf.io/vuywp/. DOI: 10.17605/OSF.IO/VUYWP.68. Publicly accessible Pseudomonas sequences were accessed via the Pseudomonas Genome Database (https://www.pseudomonas.com/) and the SBW25 genome assembly was accessed via NCBI (NCBI Assembly: ASM922v1, GenBank sequence: AM181176.4).

Field-specific reporting

Please select the one below that is the best fit for your research. If you are not sure, read the appropriate sections before making your selection.

☐ Life sciences ☐ Behavioural & social sciences ☒ Ecological, evolutionary & environmental sciences

For a reference copy of the document with all sections, see nature.com/documents/nr-reporting-summary-flat.pdf

Ecological, evolutionary & environmental sciences study design

All studies must disclose on these points even when the disclosure is negative.

Study description	This manuscript outlines an experimental evolution study which involved evolving independent clonal replicates of non-flagellate Pseudomonas fluorescens variants on soft agar motility plates. We recorded the time-to-emergence of the motility phenotype, and for a subset of our samples subsequently determined which first-step mutation resurrected flagella-mediated motility using Sanger or whole-genome sequencing. For a subset of these evolved motile lines, we additionally performed fitness assays to assess levels of pleiotropy and the strength of the motility phenotype.<br><br>Biological replicates of clonal samples for the evolution experiments were formed by making a fresh streak plate from a cryopreserved clonal stock and taking one distinct colony per independent replicate. We ensured that a minimum of three biological replicates were used for each distinct condition when performing fitness assays. We compared multiple variables within the study, namely the strain background, environment, mutational target, and strength of the resultant motility phenotype.<br><br>We searched for a gene by environment interaction using a Kruskal-Wallis chi-squared test on the observed mutational targets realised during selection. Our explanatory variables were environment, which was a discrete factor of 4 levels (LB, M9, M9+Gln, and M9+Glu), and strain background, a discrete factor of 2 levels (SBW25 ΔfleQ and AR2). The tally of observed mutational targets observed within each condition were divided by the sample size to form the discrete response variable 'proportion of mutant lines'.<br><br>We then compared motility phenotypes using the response variable of relative migration area (48 h); (calculated as [the averaged migration area of the 'test' mutant]/ [the averaged migration area of the common mutant (ntrB A289C)]). A Kruskal-Wallis post-hoc Dunn test was used to assess changes to the response variable across strain backgrounds, a discrete explanatory variable of 4 levels (A289C, Δ406-417, A683C, and glnK Δ84.). This test was performed for each of the four 4 environments independently, as we were only interested in intra-environment differences. A similar approach was used for assessing levels of pleiotropy exhibited by evolved mutant lines. In this test, each of the 4 environments were treated independently and the tested strains were treated as a discrete explanatory variable of 3 levels (Ancestor (AR2), A289C, and Δ406-417). The response variable in this case was 'relative growth rate vs AR2 ancestor', which was an area under the curve calculation made for each strain and made relative to the area under the curve of the AR2 ancestral line. A one-way ANOVA post-hoc Tukey HSD test was used for this analysis.<br><br>Finally, we compared the distribution of time-to-emergence of the motility phenotype between a genetically augmented line and their ancestor, an explanatory variable of two levels (AR2 and AR2-sm). The response variable was a continuous distribution, which was made by tallying the number of replicates that achieved motility on a given day, and subsequently dividing these numbers by the sum of lines that evolved across the experiment to get a daily proportion. A Wilcoxon rank sum tests with continuity correction was performed for two environments independently, as we were only concerned with intra-environment variation between the two strains. This test was repeated twice for two independent pairs of strains (AR2 and AR2-sm; Pf0-2x and Pf0-2x-sm6).<br><br>When assessing mutational spectrum across conditions, our sample sizes varied from 5 to 25 samples per condition in SBW25-derived lines, with the higher sample sizes weighted in favour of M9 minimal medium, which was the primary environment used in the study. A range of samples across conditions ranged from 6 to 29 in Pf0-1-derived lines, with the number of samples weighted toward Pf0-2x ancestral lines. These sample sizes were appropriate for our purposes owing to the clear pattern in mutational preference, which was robust across both strain and environment.
Research sample	Two ancestral strain backgrounds were used in this study, both derived from the soil bacterium Pseudomonas fluorescens. An immotile variant and a sliding-motility variant were derived from strain SBW25, which has a documented annotated genome (NCBI Assembly: ASM922v1, GenBank sequence: AM181176.4). An additional immotile genetic background was derived from strain Pf0-1, which for the purposes of this study was genetically analysed on a locus-by-locus basis (using https://www.pseudomonas.com/). These two genetic backgrounds were chosen as they had been documented in a previous study to evolve motility in similar (targeting the same regulatory pathway) but distinct (one evolved in parallel and the other did not) ways (Taylor et al., Evolutionary

resurrection of flagellar motility via rewiring of the nitrogen regulation system. (2015). Science. 347(6225):1014-7). The SBW25 and Pf0-1 derivatives were each isolated and cryopreserved in clonal samples, and the cryopreserved stocks were used to seed indpendent replicates during evolution experiments.

Sampling strategy

During sampling, motile strains were isolated from the leading edge of the bacterial growth by patching an inoculating loop's worth of leading cells onto fresh 1.5% agar. This allowed us to dilute and separate the cell sample to isolate a single colony forming unit per sample, which was treated as being founded by a single cell (standard microbiology practice). This was subsequently treated as one independent replicate. This technique introduced two key bottlenecks: we only took from the leading edge and only isolated one motile colony forming unit per sample. Both of these bottlenecks are addressed within the manuscript.

Additionally we only sequenced a subset of the evolved lines, and as such the sample size for each independent condition (environment/unique strain background) ranges from 5 to 25 samples in SBW25 lines and 6 to 29 in Pf0-1 derived lines. Sample sizes different across strain backgrounds and nutrient regimes, with larger sample sizes chosen for Pf0-1 derived strains so that we could capture the diverse mutational spectrum of mutational routes, and weighted in favour of the M9 environmental condition in SBW25 derived strains, as this was our "primary" nutrient regime used in the study (this regime is included in all 5 figures of the main manuscript). We observed a strong statistical effect with the included sample sizes owing to a clear signal in the data which either heavily favored identical genetic outcomes or diverse genetic outcomes dependeding on just one explanatory variable. As such our current sample size is sufficient for the conclusions drawn from the data.

Data collection

All data was collected directly by the lead author of the manuscript (J. S. Horton) or co-author L. M. Flanagan in the same laboratory.

Time-to-emergence data was recorded daily after visually inspecting the motility plates for emergent motile zones. Invasion assay data included in the raw data files was determined by leaving independent replicates to grow for 48 h before photographing the plates using a Canon G10 camera in a mounted light-proof box. This equipment ensured that photos were captured from a standardised overhead position and that outside lighting would not affect the identification of motile zones. The plate images were subsequently analysed manually using Powerpoint (Microsoft Office 365) and the surface area was calculated (A=πr^2) using an average of two measurements of r, which were drawn laterally and longitudinally.

Mutation data was primarily collected by analyzing Sanger sequencing data (performed by Eurofins Genomics). The clipped sequencing files provided by Eurofins Genomics were aligned against the native locus sequence (which was downloaded from https://www.pseudomonas.com/) using nBLAST (https://blast.ncbi.nlm.nih.gov/Blast.cgi). Called SNPs or indels were only confirmed if the provided chromatogram for the query sequence (also provided by Eurofins Genomics) called the change with high confidence. If this was in any doubt, the sequencing was repeated. Full-genome Illumina sequencing was conducted by the Milner Genomics Centre, Bath, UK and MicrobesNG, Birmingham, UK. Microbes NG reads reported high coverage (>30 per base mean), however some samples from the Milner Genomics Centre, Bath, UK reported mean coverage per base at <10. As such we performed Sanger sequencing on these samples to confirm any predicted mutations. In these instances all Sanger sequences matched those reported by Illumina sequencing. Illumina data was analysed and mutations called using Snippy (https://github.com/tseemann/snippy).

Microbial growth kinetics were recorded autonomously using a Multiskan™ FC Microplate Photometer (Thermo Fisher Scientific). Area under the curve data was calculated per independent replicate from this data (area under the curve data is provided in the raw data files) using the Trapezoidal Rule (see https://bit.ly/3njzXCU).

Timing and spatial scale

Motility assays: Samples were evolved on 88mM petri dishes containing soft agar (0.25%) and monitored at least once daily for periods spanning up to 11 days. As it was not practical to autonomously monitor the number of plates used in the study and it was not possible to manually monitor the plates throughout out-of-work hours, we opted to record the day of emergence rather than a specific hour interval. We sampled motile cells as soon as a motile zone was observed (typical radius of motile zone when sample taken = 0.5 cm) which was 0 h < and < 24 h from time of emergence. We opted to terminate the evolution experiments after 11 days had elapsed, owing to drying of the motility agar in the incubator. This would have hampered swarming motility in emergent lines after this date and therefore may have either prevented evolution of motility, or slowed the phenotype emergence significantly enough to skew the time-to-emergence data.

Microbial growth kinetics: Autonomous readings were recorded every 10 minutes for a total period of 24 h. This time was chosen as populations growing in lysogeny broth reach stable stationary phase over this period but do not yet descend into their death phase, which would affect comparisons across strains and environments using area under the curve.

Invasion assay: We allowed two strains to compete for a total period of 24 h. This time was chosen as all samples were monitored and sampled within 24 h of emergence (see above). Therefore this time point allowed us to mimic the original conditions as closely as possible. Samples were taken from the leading edge at hour 24 of the assay.

Race assay: Independent replicates were allowed to grow for 48 h on 88mM petri dishes containing soft agar (0.25%) prior to imaging. 48 h was chosen as an optimal time point for this assay as leaving plates for longer than 48 h increased the likelihood of mutants acquiring an additional mutation and becoming established at the leading edge and forming a 'bulge', which distorted the concentric circle motile zone and meant that the replicate had to be discarded from the study. Leaving plates for less than 48 h, however, meant that we would be less able to capture small differences in surface area coverage between strains, which would have been lost in the error during measurement (see above). Therefore 48 h was chosen as an appropriate time point to conduct the assay.

Data exclusions

Data were excluded from the study on two conditions: contamination or unwanted additional mutation. This latter element only came into effect during the race assay (see above) wherein the purpose was to evaluate the motility phenotype granted by the founding populations of the plates. Contaminated plates were observed rarely during evolution experiments, and if observed where discarded from the study and not included in the data set, as co-evolution with another species may have affected the adaptive dynamics.

Reproducibility

All tests were found to be reproducible, as all assays aside from the invasion assay were completed independently at least twice. This includes rounds of evolution experiments to increase sample sizes as well phenotype comparison assays. During these tests we

observed no contradictory results. For full transparency we have plotted independent replicate data points instead of error bars within the manuscript, which demonstrate the consitency in our results.

Randomization

Experimental bias was controlled for using randomization whenever necessary, which in the case of our manuscript was during evolution experiments and race assays. We observed prior to the study that there is slight batch-to-batch variation in each batch of soft agar (from which 12 plates can be poured). As this may have affected the migration rate of emergent zones, when >12 plates were poured per condition we assigned plates to strains at random. Soft agar was also used during the race assay, but batch-to-batch variation was controlled for by splitting strains evenly across the batches of agar. This was achievable as we analysed no more than 4 lines at one time, allowing us to assign 3 biological replicates per line to each batch of agar (4*3 = 12).

We also randomised the subset of independent lines sent for sequencing. As described above, we intentionally introduced an artificial bottleneck during sampling of emergent motile zones, as we always picked from the leading edge. The spread plates that were streaked from these picks formed individual colony forming units, of which one was picked at random. As these colonies had homogeneous morphologies, there was no deliberate way for us to introduce a bias in the colony chosen for sequencing.

The other assays performed for this study, namely characterising different phenotypes of strains under various nutritional contexts, required no randomisation measures. These assays were performed using ≥3 independent biological replicates, which were subsequently collated for data analysis. As such no biases could be introduced throughout these assays.

Blinding

Blinding was unnecessary in our study owing to the major results being reliant on SNPs and small indels provided by sequencing data, wherein the result is immune to bias as no interpretation is involved. We were unable to determine the genotypes of motile mutants from the emergent motile zones prior to sequencing as each motility phenotype and colony morphology is visually highly similar. As we always sequenced the samples before conducting any additional phenotypic analysis, we were unable to introduce bias prior to determining the genotype of the samples. Additionally, as our manuscript shows, after conducting analysis of phenotypes some unique mutations remain indistinguishable phenotypically, meaning we would be unable to be selective regarding which samples were sent for sequence analysis.

Did the study involve field work? ☐ Yes ☒ No

Reporting for specific materials, systems and methods

We require information from authors about some types of materials, experimental systems and methods used in many studies. Here, indicate whether each material, system or method listed is relevant to your study. If you are not sure if a list item applies to your research, read the appropriate section before selecting a response.

Materials & experimental systems

n/a	Involved in the study	
☒	☐	Antibodies
☒	☐	Eukaryotic cell lines
☒	☐	Palaeontology and archaeology
☒	☐	Animals and other organisms
☒	☐	Human research participants
☒	☐	Clinical data
☒	☐	Dual use research of concern

Methods

n/a	Involved in the study	
☒	☐	ChIP-seq
☒	☐	Flow cytometry
☒	☐	MRI-based neuroimaging
